# P-1420. Improving Immunization Rates among Underserved Pediatric Patients through a Student Run Free Clinic's Successful Public Health Partnership

**DOI:** 10.1093/ofid/ofaf695.1607

**Published:** 2026-01-11

**Authors:** Amisha Chaudhary, Beatrice Russell, Andrew Ji, Grace Oh, Emily Fleck, Abdul Malik, Cahil Potnis, Ingrid Camelo, Juan Rivera Salva

**Affiliations:** Medical College of Georgia, Augusta University, Augusta, GA; Medical College of Georgia, Augusta University, Augusta, GA; Medical College of Georgia, Augusta University, Augusta, GA; Medical College of Georgia, Augusta University, Augusta, GA; Medical College of Georgia, Augusta University, Augusta, GA; Medical College of Georgia, Augusta University, Augusta, GA; Medical College of Georgia, Augusta University, Augusta, GA; The Johns Hopkins University School of Medicine, Baltimore, Maryland; Medical College of Georgia, Augusta University, Augusta, GA

## Abstract

**Background:**

Early childhood vaccinations are critical for preventing severe illness and reducing community spread. Despite longstanding public health efforts, significant coverage gaps remain—especially among uninsured, underinsured, and impoverished children. Asociación Latina de Servicios del CSRA (ALAS) Pediatric Clinic, a Student-Run Free Clinic (SRFC) partnered with the Medical College of Georgia, provides free healthcare to uninsured children from families 200% below the federal poverty line. To address immunization barriers, the clinic partnered with the GA Department of Public Health (DPH) to offer free vaccines, administered by trained medical students. This study assesses immunization changes after clinic visits.

**Figure:**
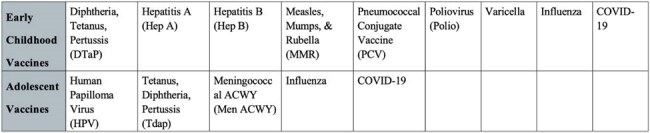

**Figure:**
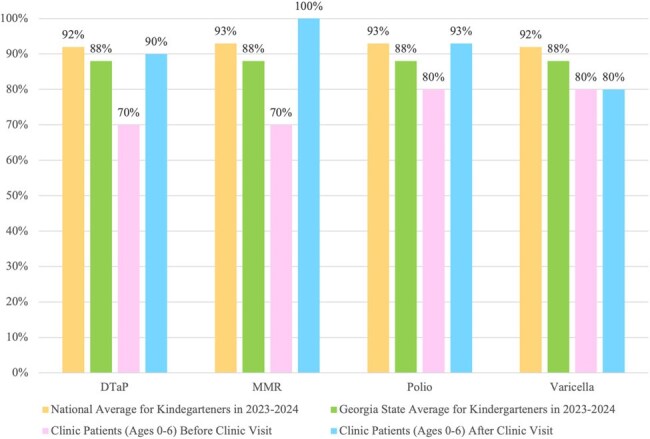

**Methods:**

This study analyzes 25 patients seen since January 2025, when vaccine administration began through the DPH Vaccines for Children program. Eligibility was determined using the Georgia Registry of Immunization Transactions & Services per CDC guidelines. Patients were offered due vaccines during their visit. Included vaccines (Table 1) followed CDC recommendations (excluding RSV, Rota, Men B, Dengue, and Monkeypox). Vaccination status was recorded before and after appointments, regardless of vaccine acceptance.

**Figure:**
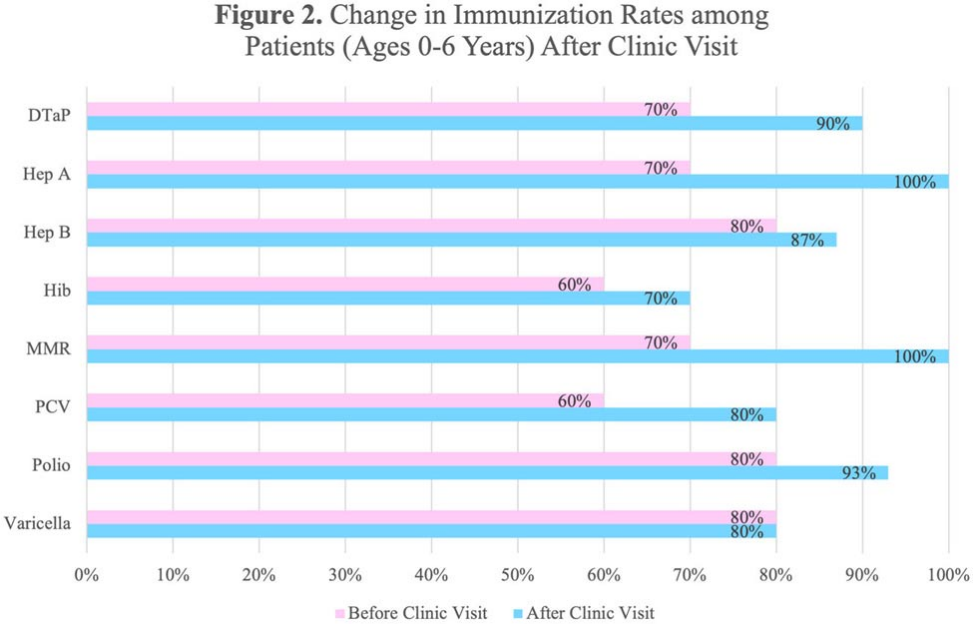

**Figure:**
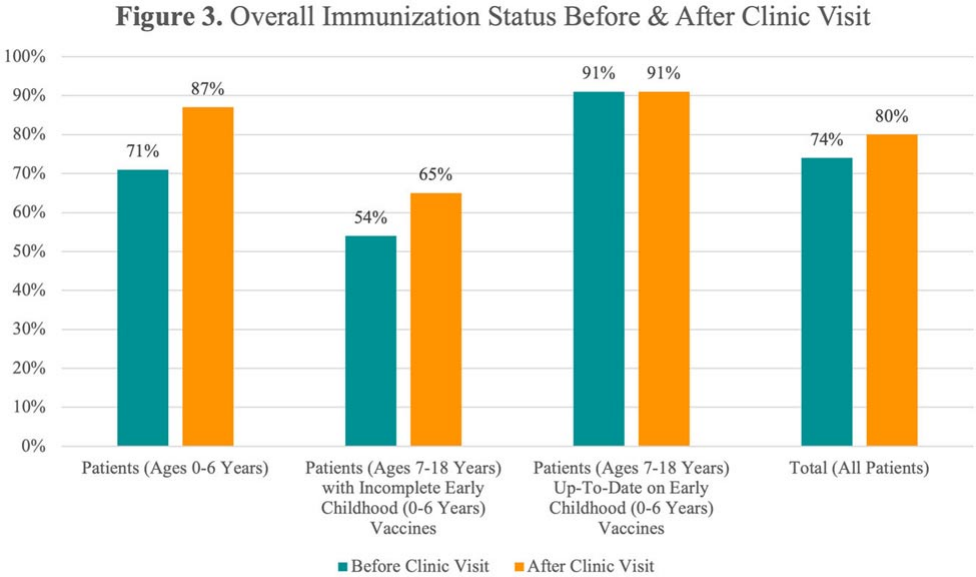

**Results:**

Before clinic visits, patients aged 0-6 years had lower immunization rates than 2023-2024 national and state kindergarten averages (Figure 1). After visits, coverage improved significantly, with 100% MMR and Hep A coverage in this age group (Figure 2). Overall, both age groups saw increased immunization coverage post-visit, especially for early childhood vaccines (Figure 3). Data collection is ongoing.

**Conclusion:**

These findings reveal large immunization gaps among underserved pediatric patients and support SRFC in-clinic vaccination as an effective solution. SRFC partnership with the DPH increases access to essential vaccines, improving coverage in underinsured children. Previously, patients required separate DPH visits for vaccines, burdening families facing transportation, language, and documentation challenges. In-clinic vaccination and interpretation services reduce barriers and foster relationships that support patient education and decision-making.

**Disclosures:**

All Authors: No reported disclosures

